# Peeking and lying in the temptation resistance paradigm in 2.5-year-olds: The role of inhibitory control

**DOI:** 10.1371/journal.pone.0278099

**Published:** 2022-12-07

**Authors:** Marta Białecka-Pikul, Arkadiusz Białek, Małgorzata Stępień-Nycz, Victoria Talwar, Sandra Bosacki

**Affiliations:** 1 Jagiellonian University, Krakow, Poland; 2 Nicolaus Copernicus University, Toruń, Poland; 3 McGill University, Quebec, Canada; 4 Brock University, St. Catharines, Canada; National Center for Child Health and Development, JAPAN

## Abstract

The main aim of the present study was to establish whether inhibitory control (IC) abilities influence the peeking and lying behaviours of 2.5-year-olds, as measured by a modified temptation resistance paradigm (mTRP). Using a longitudinal design, 252 children’s IC abilities were tested at ages 1.5, 2 and 2.5, as well as their ability to lie at age 2.5. Results showed that 35% of 2.5-year-olds peeked, 27% of peekers lied and 40% of non-peekers falsely confessed they had peeked. Non-peekers had higher IC than peekers at ages 2 and 2.5. Lower IC at age 2 increased the probability of peeking at age 2.5 by 6 times. The highest level of IC was presented in children who followed the adult’s restrictions in the mTRP and were then able to tell the truth about their behaviour. These results suggested that the first, or so-called primary, lies of 2.5-year-olds are probably spontaneous, rather than deliberate. Implications for further research were discussed.

## Introduction

Parents report that children begin telling their first lies as early as 2.5 years of age [1, 2]. Importantly, lying is understood here in the same way as it is in psychological literature, namely as the ability to intentionally and verbally deceive others [3]. In other words, it is the ability to deliberately instil a false belief in another person mostly for self-serving purposes such as to conceal a transgression [4]. On the one hand, the deliberateness of these first, or primary, lies of such young children should be reconsidered when taking into account that prior to the age of 4 years, most children are unable to reason about the false beliefs of others as they do not pass the explicit false belief test [5].

On the other hand, concealing one’s own transgressions is a very cognitively demanding skill, as it requires self-regulating or inhibitory abilities that are only beginning to develop in toddlers [6]. Considering these two issues, it seems paradoxical that the most typical experimental procedure used to measure young children’s lies is the temptation resistance paradigm (TRP) [7]. To pass this kind of experimental procedure, strong inhibitory skills are probably needed because in the TRP, a child is left alone and is forbidden to take a particular action (e.g. peek under the cover) in a tempting situation (e.g. when an interesting, new sound is elicited from under the cover, and the child was previously encouraged to peek and guess the name of the hidden toy). When the researcher moves back and asks, ‘*Did you peek*?’, the child may confess to peeking (say ‘*yes*’) or lie (say ‘*no*’). Therefore, from a methodological point of view, peeking behaviour or impulsive action that transgresses the rule in the TRP is a first and necessary condition that allows the researcher to measure children’s lies. Moreover, as far as the results are interpreted, studies have found that 80% of 2- to 3-year-olds peeked, and about 30% of peekers lied about it [8–10]. Thus, many researchers claim that some children first react impulsively and then reflectively and deliberately conceal their transgression. This seems doubtful, as children are supposed to demonstrate opposite regulatory abilities in the same situation.

We attempt to disentangle this paradox using a longitudinal study design. In this study, we asked whether earlier, i.e. observed at ages 1.5 and 2, and simultaneous, i.e. measured at age 2.5, inhibitory control (IC) abilities influenced the peeking and lying behaviours of 2.5-year-olds, as measured by a modified TRP. Inhibitory control, i.e. ‘the ability to inhibit responses to irrelevant stimuli while pursuing a cognitively represented goal’ ([11], p. 1033), is considered a key component of a much broader construct of self-regulation (see [12]), and it is also an important dimension of executive functioning or—in reference to toddlers—of effortful control [6, 11, 13]. Below, we first review the studies on IC in toddlers and then refer to results obtained directly with the TRP in this age group, and finally, we focus on how peeking and lying in the TRP are related to children’s IC skills.

### Inhibitory control skills during toddler years

Inhibitory control (IC) is an executive functioning skill [14] and may be defined as the capacity to suppress inappropriate approach responses under instruction [15]. Inhibitory control, or response inhibition, develops rapidly during the toddler and preschool years [6]. Inhibitory control markedly improves between ages 2–6. For this age group, IC is usually assessed by measuring the child’s ability to delay, temper or suppress impulsive responses evocated by a task [11]. One example of such a task is the Snack Delay Task [15], in which a child has to wait for the researcher to ring a bell before retrieving an M&M from underneath a glass cup. This is an appropriate measure for first expressions of inhibition—precisely delay inhibition—in 2-year-olds. According to Garon, Bryson and Smith [16], this kind of simple response inhibition paradigm has minimal working memory demands, in which a child is given the opportunity to delay or withhold a prepotent response. Therefore, it is most suitable for the youngest children.

The complex inhibition [16], or so-called conflict tasks [11] have greater working memory demands. This higher cognitive load is due to the fact that children are asked to inhibit an action in reference to one stimulus and also to behave in a particular way in reference to another, sometimes conflicting stimulus. For example, in the Go/No-Go Task, participants are asked to press a button in response to a visually presented picture, but different signals occasionally occur that they are instructed to ignore [14]. The Animal Go/No-Go Task is also based on this paradigm and adapted for young children [17]. This task, as it is more difficult, is also more suitable for children older than 2 years, e.g. 2.5-year-olds.

Therefore, to describe the development of IC skills in toddlers, we need to measure them in a valid way—in other words, using age-sensitive tasks. Based on the Petersen et al. [18]) analysis of 198 studies using different measures of IC, we propose that behavioural manifestations of IC change across development. These changes are consistent with the notion of heterotypic continuity, namely that IC skills may appear differently in younger and older children. Thus, we designed a longitudinal study to measure IC skills using a delay inhibition task with 1.5- and 2-year-olds and using conflict inhibition with 2.5-year-olds.

### Temptation resistance paradigm as a measure of lying behaviour

In general, researchers use the temptation resistance paradigm (TRP) to study children’s lies [3, 7, 9, 19, 20]. The TRP involves a series of trials that allow children to play with a target toy. These are followed by a prohibition trial, during which the researcher is absent or turns away after instructing the children not to touch or look at a toy under a cover (or behind their back). This prohibition trial of the TRP is the most important point of this paradigm because during this trial, children have the opportunity to transgress the rule (i.e. to peek at the forbidden object). Only after the child’s activity that follows the direct request to inhibit their behaviour—in other words, due to this transgression—can the researcher ask the main test question of the TRP: ‘*Did you peek*?’ This question allows participants to either lie (say ‘*no*’ after peeking) or to tell the truth about their behaviour. Then, the researchers can calculate the number of non-peeking children and, more importantly, the number of peeking children, who fall into two groups: liars and truth-tellers [9, 20, 21]. Such a paradigm provides researchers with the opportunity to elicit children’s spontaneous lies through a procedure that appears to be ecologically valid and naturalistic for 2–3-year-olds [3, 20, 22].

Using this paradigm, researchers have charted the development of children’s lie-telling from toddlerhood to early adolescence (e.g. [7–9, 20, 23–26]). It turns out that among 2-year-old children, 20–30% of those who peeked lied, and in 4-year-olds, it was about 50–60% of the children. After the age of 5, more than 80% of peekers presented false statements when asked whether or not they had looked at the toy during the researcher’s absence (see [3] for review). Moreover, based on these descriptive data, Talwar and Lee [9] propose distinguishing three types of verbal deceptions: primary lies (in children before age 4), secondary lies (in children ages 4–7) and tertiary lies (in children older than 7). Importantly, the authors point out that primary lies might or might not be intentional attempts to induce false beliefs. That is, a toddler’s lies, as measured by the TRP, could also not be considered lies in accordance with the standard definitions of ‘lie’ (see [19]), even when the authors use the term ‘lies’ (or sometimes ‘spontaneous lies’). Here, we should mention that Stern and Stern in 1909 [27] coined the term ‘pseudo-lies’ to describe the initial deceptions produced by young children, which are mistaken utterances or ‘wish fulfilment’, rather than truly deliberate instances of deceitfulness [9, 28, 29]. We may also refer to these initial lies as “deceptions-in-action” [30], aimed at achieving a situation favourable to oneself. This first signs of deceptions may be intended to affect others’ behaviour and not necessarily others’ beliefs.

Bearing in mind this controversy (whether primary lies are real lies or pseudo-lies), it is important to admit that it was Evans and Lee [8] who used a modified TRP to measure lie-telling in this youngest group of children, i.e. 2–3-year-olds. First, unlike previous studies in which a researcher leaves the room for a moment (i.e. providing an opportunity for the child to peek), the researcher in their study remained in the room but turned their back to the child. This allowed the child to peek when the researcher was not looking in the child’s direction while also accommodating the paradigm to the young age of the children who may not be able to stay alone in the room. Second, Evans and Lee [8] claim that ‘consistent with the hypothesis, we established experimentally that 2-year-olds will spontaneously tell lies’ (p. 1961). Third, they also state that it was found that after controlling for age, 2–3-year-old children who performed better on executive functioning tasks (in a conflict inhibitory task, i.e. shape Stroop) were significantly more likely to lie. Finally, Evans and Lee [8] state, ‘due to the low frequency of non-peekers in the present study, we were unable to test the possibility of a “yes” bias’ (p. 1961) as the explanation for why 75% of 3-year-olds honestly confessed their transgression.

Overall, this last issue is extremely important because it supports the idea that toddlers’ low inhibitory skills might be responsible for the high rate of peeking in the TRP and also for the low rate of peekers–liars. Importantly, to the best of our knowledge, to date, previous research has only examined children who peeked due to a lack of non-peekers. For example, Williams et al.’s [10] study found 58 peekers vs. 7 non-peekers; Leduc et al.’ [31] study found 63 peekers vs. 4 non-peekers; whereas Evans and Lee’s [8] study found 52 peekers vs. 13 non-peekers. We claim that to fully understand the emergence of lie-telling in this primary stage, it is also important to examine those children who abstain from peeking. Such a focus allows us to compare the cognitive characteristics of peekers and non-peekers, especially their IC skills. Moreover, it allows us to ask whether all such young non-peekers admit the truth for the ‘*Did you peek*?’ question, or are there children who—maybe according to the ‘*yes*’ bias [32, 33]—admit they peeked even if they did not. Thus, the non-peeking children are an interesting group because it would seem that they comply with the rule of not peeking. Perhaps non-peekers do not transgress the rule because, as rule followers, they have high inhibitory skills and are more prone to comply with an adult’s restrictions and expectations.

### Peeking and lying behaviour in young children and their relations to inhibitory control skills

Relations between IC and lying are clear in tasks that include ‘deceptive pointing’, in which children may deceive by pointing to a misleading location. For instance, Sodian and colleagues [34] analysed data from studies in which participants watched while a reward was hidden in one of two opaque containers. Participants were then asked to show one of the containers to a competitive partner (e.g. a robber), who would keep the reward for himself or herself. Three-year-old children typically failed to misinform the competitor, but 4-year-olds have been found to act spontaneously and have no problem with deception. Carlson and colleagues [35] also found that the source of the 3-year-olds’ difficulty with deceptive pointing possibly stems from a failure to inhibit their behaviour. Moreover, Evans, Xu and Lee [36] showed that 4-year-olds’ ability to maintain consistency (not allow for semantic leakage) during the TRP was positively related to IC skills. Thus, based on these studies, in older 3- and 4-year-olds, who have more advanced inhibitory skills than 2- to 3-year-olds, deliberate lying might be present. Once more, we suggest that due to their emerging inhibitory abilities, younger children are less able to inhibit, and their behaviour might be more spontaneous and impulsive. Thus, their lies might also not be so deliberate. This supposition remains in accordance with the results of meta-analysis on the relation between lying behaviour in children and their executive function [37] which indicated that (1) the size effect of the relation between lying and EF was significant, but small, and (2) the correlation of executive functions with children’s initial lies was significantly smaller than with children’s ability to maintain lies.

This claim is also in accordance with the non-coherent pattern of results of previous studies in which the TRP paradigm was used with toddlers. As mentioned above, Evans and Lee [8] found that among 2.5-year-olds, only conflicting IC skills (Stroop Task), but not delay inhibition (Gift Delay Task), predicted lying ability. Moreover, Williams et al. [10] found that higher levels of inhibition (Whisper Task) and planning (Kitten Delivery Task) abilities were found in peekers–liars, compared to same-aged peekers–non-liars. Similarly, Leduc et al. [31] showed that children around 3 years of age, who were more able to inhibit their reactions (Whisper Task) were more likely to display lying behaviour in the TRP. However, to date, no studies have shown that the IC skills of non-peekers are comparable to those of peekers in this age group, although no differences were found in older (4-year-old) children [36]. Thus, we cannot exclude the possibility that non-peekers’ compliance with an adult might be based on their strong ability to inhibit their impulses, and thus this group might present the highest level of IC.

### Current study

The data presented here examined the influence of IC skills on the peeking and lying behaviours of children younger than age 3 in a TRP. To measure peeking and lying or truth-telling behaviour, children participated in a TRP similar to that of Evans and Lee [8]. Importantly, the longitudinal design of the study allowed us to assess IC skills three times in the same group of children, and we measured them twice—before peeking and lying were assessed—and also once at the same age, i.e. at the age of 2.5.

Based on previous studies (e.g. [8]), it was expected that most 2.5-year-olds would peek at the forbidden toy. We also predicted that the highest level of IC abilities would be present in 1.5-, 2- and 2.5-year-olds, who represent the group of non-peekers, in comparison to peekers at the age of 2.5. In other words, we expected that lower levels of inhibition should be present in children who do not follow the rules in the TRP (i.e. peekers), compared to those who follow them (i.e. non-peekers). This result seems plausible based on previous data on the inhibitory abilities of such young children (e.g. [35]). Moreover, if lower levels of IC are observed in 2.5-year-old peekers, their behaviours and their answers—also for the ‘*Did you peek*?’ question—should be quick and spontaneous. Therefore, the IC of both groups—liars and non-liars—might not differ. Additionally, we plan to explore the question of whether all non-peekers truly admit they did not peek.

## Method

### Participants

This study was part of a larger research project *Birth and development of mentalising ability*, conducted in the Institute of Psychology, Jagiellonian University. Children visited the laboratory six times (every 6 months). Participants first visited the lab at 12 months old (the first time point, T1), and they visited for the last time when they were 42 months old (the sixth time point, T6). It should be noted that during the first lab visit, we tested 357 1-year-olds, and during the last lab visit, we tested 299 3.5-year-olds. The majority of the children were from urban areas within Poland (80%); the majority of the children’s parents were educated at the university degree level (76%), and almost all of them spoke only Polish to their children (99%).

The data presented in this paper refer to the second (T2), third (T3) and fourth (T4) time points. During the second time point (T2), 351 children, aged approximately 1.5 years old (*M* = 80.19 weeks, *SD* = 1.9 weeks, 159 girls), were tested. At T3, which took place half a year later, the children were 2 years old (*N* = 343; *M* = 104.28 weeks, *SD* = 1.68 weeks, 151 girls), and at T4, they were 2.5 years of age (*N* = 325; *M* = 129.4 weeks, *SD* = 1.5 weeks, 143 girls). We need to admit that at T4, the data from our modified TRP were coded for 252 children out of the 325 tested children (for 73 children we did not obtain data for the mTRP), and thus our final group consisted of *N* = 252 children (at T4 *M* = 129.36 weeks, *SD* = 1.44 weeks, 116 girls). Below, we analyse the results obtained only for this group also at T2 and T3. It should be noted, that some of these children have missing data at different tasks or did not complete a full follow-up at all time points, therefore we provide exact *n*s for all analyses. In the case of 73 children, no data were obtained for the modified TRP (mTRP) due to various technical problems with video or sound recording (*N* = 7), a parent interrupting (*N* = 6), the researcher making a mistake when giving instructions (*N* = 5), lack of time due to the lab visit being delayed (*N* = 4) or a child’s refusal to cooperate (*N* = 51). Importantly, children with no data for the mTRP task did not differ from the rest of the children according to the results of IC measures at all time points, i.e. at T2, T3 and T4 (all *p’s* >.05).

### Procedure

The present study was carried out as a large, 3-year longitudinal research project that received clearance from the Research Ethics Committee of the Institute of Psychology, Jagiellonian University. The parents and children were recruited on a voluntary basis via personal advertisements. Informed written consent was received from all caregivers prior to the study. Children received a small gift at the end of each lab visit. Each visit to the laboratory lasted roughly 1 hour on average, with 22–28 short tasks that measured different socio-cognitive and cognitive abilities. Precise descriptions of all these tasks are not presented here because we do not refer to their results. Importantly, we also checked whether the task order influenced any task results, and no effect was found for the results of the tasks presented below (all *p*s > .05).

The lab sessions took place in the same small room, and the child always sat at a table on a parent’s lap or alone (but the parent was behind the child), with the researcher in front of or close to the child. The parents were instructed to remain silent and neutral throughout the sessions. At T2 and T3 all children stayed in the room with their parents. At T4 30% of children agreed to stay in the room alone, with parents waiting in the second room. Short breaks occurred between the tasks when needed by the child. The entire lab session was recorded by two video cameras (CCTV).

### Measures

#### Early inhibitory control tasks (T2, T3, T4)

At T2 and T3 (i.e. for the 1.5- and 2-year-olds), we used the Snack Delay Task, which assesses only the basic ability to delay response. At T4 (i.e. for 2.5-year-olds), we used the Animal Go/No-Go Task, which assesses the ability to suppress one reaction and initiate another.

In the Snack Delay Task [13], the child sat on the caregiver’s lap, and the researcher at the opposite end of the table placed a crisp on a plate and covered it with a transparent plastic cup. Then, the researcher asked the child, ‘*Do you like crisps*?’ and, after receiving a positive answer, said, ‘*You have to wait for this crisp*. *I have to leave the room now*, *but as soon as I get back*, *I’ll give you this crisp*’. The researcher returned after 60 seconds (at T2) or 90 seconds (at T3), and the time of the delay was measured as an index of IC skills. The coders’ agreement for the time of delay at T2 and T3 was *r* = .87 and *r* = .98 (both *p*s < .05), respectively.

In the Animal Go/No-Go Task [17], the child sat close to the researcher and viewed a laptop monitor with animals (a dog, cat and frog) appearing on it. After the instruction, ‘*Press this red button when you see a dog or a cat*, *but not when you see a frog*’, and after two test trials (the researcher could repeat the instructions and praise the child), a series of 11 trials (5 no-go) were conducted. The number of inhibited actions was calculated as an index of IC skills. As 30% of children agreed to stay in the room alone and 70% stayed with their parent present, we compared these two groups and found no differences in their scores in the Animal Go/No-Go task (*F*_(1,104)_ = 0.02, *p* = .876).

#### Modified temptation resistance paradigm (mTRP, T4)

**Fig 1:**
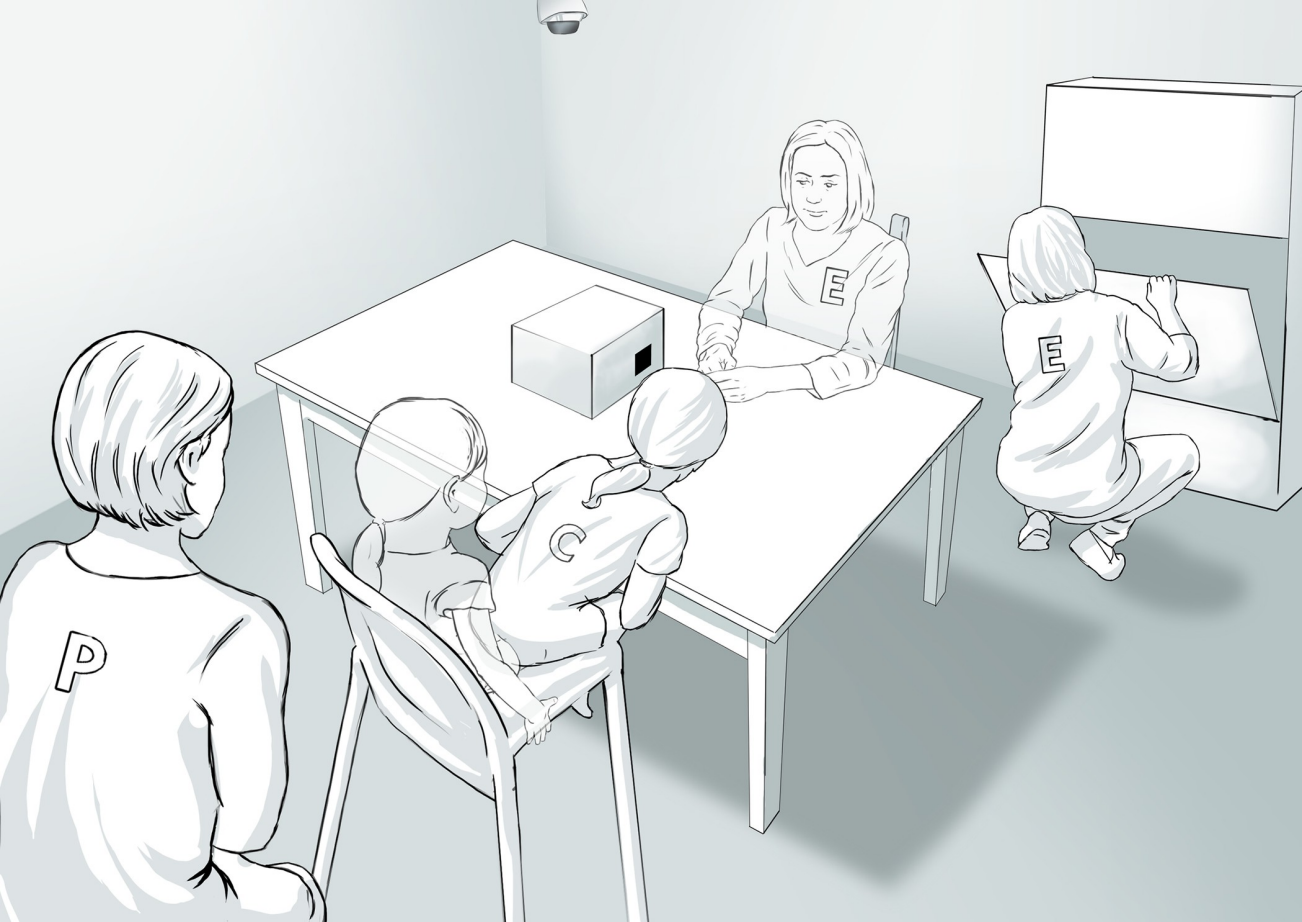
Modified temptation resistance paradigm. Fading figures of a child and a researcher present their position before the prohibition trial.

Following Evans and Lee [8], we prepared a modified version of the TRP (mTRP). In the mTRP, the researcher sat on the opposite side of the table from the child. First, with her hands under the table, the researcher played a sound (e.g. a dog barking) and—hidden from the child’s view—placed a toy dog in the box (20 cm in height, 45 cm in length and 30 cm in width, with two 5 cm x 5 cm slots on the shorter sides) and then put the box on the table (see Illustration 1 for fading figures of a child and a researcher). Then, the researcher asked the child ‘*What do you think is inside*?’ and allowed the child to peek through the slots or lift the box if she wanted. When the child responded, the box was lifted, and the child was free to touch the toy for 10–20 seconds. In the second phase, the same procedure was repeated with a toy cat. Next, the researcher played a different (unrecognisable) sound and placed the box on the table, stating, ‘*I have to find something in the cupboard*. *Wait a minute*. *Don’t peek and don’t touch the box*’. Then, the researcher turned around and went to the corner of the room, where she squatted in front of a cabinet with her back to the child to ensure that the child could not see her face (see Fig 1 for non-fading figures of a child and a researcher). After 30 seconds, the researcher returned to the child, stood still, looked at the child and then paused for 5 seconds before asking, ‘*Did you peek*?’ If the child had lifted the box and the toy was visible, the researcher did not ask any further questions; only if the box was laid over the hidden object were the following questions asked: 1) ‘*Did you peek*?’ and then 2) ‘*What was inside the box*?’

Comparing this procedure to the original of Evans and Lee [8], we made two important changes: 1) the parent stayed in the room with the child; and 2) the peeking reaction did not consist of turning around but rather lifting a box or peeking through a slot (see Illustration 1). Two reasons for these changes must be clarified. First, given the fact that in Polish culture, parents prefer to stay with such young children at all times in the same room during the testing, and children also tend to leave the room each time a parent wants to leave, we decided that the parent could stay in the room. The parents were instructed not to interrupt the procedure, even if the child attempted to avoid obeying a rule; all parents complied with this instruction. Importantly, the number of children who peeked did not differ depending on whether the child was alone (*n* = 77) or with the parent present (for *n* = 144, the parent sat behind the child; for *n* = 25, the parent sat near the child; and for *n* = 6, the child sat on a parent’s lap): for absent parents and parents sitting behind the child, χ^2^ = 0.01, *p* = .91; for absent parents and parents sitting near the child, χ^2^ = 0.44, *p* = .51.

The second change in our mTRP was the consequence of the first. That is, if a parent stayed in the room and was behind the child, we could not allow the child to turn around as they would then face the parent during the prohibition and would probably influence the peeking rate. Thus, we decided to use a box with slots to cover the toys—both in the first two trials, when the children were allowed to peek, and in the prohibition trial, when peeking was forbidden. However, what is most important, the structure of events and the questions asked in our mTRP were semantically the same as in the original Evans and Lee [8] procedure.

As for coding the children’s answers, first, we need to underline that a substantial number of 2.5-year-olds gave no answer for the first ‘*Did you peek*?’ question (*n* = 24 of peekers and *n* = 38 of non-peekers, thus 25% of the tested group). Importantly, most of these children answered the second question ‘*What was inside*?’ (only 6% of the group remained silent for this question). Therefore, when calculating the number of liars and truth-tellers among the peekers (those who lifted the box or looked through the slot), we decided to take into account the children’s answers to the second question. We believe that although in previous studies (e.g. [9]) with older children (3- to 7-years-old), the answer to this second question was the index of semantic leakage control, in contrast, for the present study, for 2.5-year-olds, it was just an expression or a denial of their knowledge in that they definitely peeked, and they had seen the bear in the box—in particular, when previously they had given no answer to the ‘*Did you peek*?’ question. Therefore, the children were coded as liars: 1) if the child peeked (i.e. looked inside the box through a slot or by lifting and putting the box back on the bear) and answered ‘*no*’ to the first question; or 2) if he or she said nothing at first and then replied ‘*I don’t know*’ to the second question. Children were coded as truth-tellers if: 1) the child peeked and answered ‘*yes*’ to the first question; or 2) if he or she remained silent in response to the first question and said ‘*a bear*’ for the second question. Using this coding procedure, two independent coders reached 94% agreement while coding 42 (16% of materials) videos.

### Analytic strategy

To achieve the goals of this study, a series of analyses were conducted. First, descriptive statistics for peeking and lying behaviours were calculated. Then, the longitudinal consistency of the inhibitory control was evaluated. Next, the groups of non-peekers and peekers—and among them, liars and truth-tellers—were compared based on their IC skills. Finally, we further analysed the role of IC in peeking and lying behaviours using multinomial logistic regression analysis. Children missing some data points were excluded from the analysis dealing with this time point but were included in other analyses, therefore the exact *n*s in each analysis differ.

## Results

### Peeking and lying behaviours

We found that in the Polish group of 2.5-year-olds, only 89 children (35%) peeked during the modified TRP paradigm. In Table 1, we present raw data on the number of children who said ‘*yes*’ or ‘*no*’ to the TRP question (‘*Did you peek*?’) and how they answered the second question (‘*What was inside the box*?’) in both situations: when they really peeked and when they did not.

**Table 1:** Number of children giving different answers in the MTRP in groups of Peekers and non-peekers.

Group	The first question: *“Did you peek*?*”*		The second question: *“What was inside the box*?*”*		
			Number of children who answer		
	Children answers	Number of children	*a bear*	Something different than *a bear* or *I do not know*	No answer
Peekers *N* = 89 (35%)	Question not asked^1^	28	n/a	n/a	n/a
	No answer	24	6^a^	12^b^	6
	*Yes*	25^a^	8	15	2
	*No*	12^b^	2	10	0
Non-peekers *N* = 163 (65%)	No answer	38	1	33	4
	*No*	60	5	53	2
	*Yes*	65^c^	8	56	1

Note

^1^ The question „*did you peek*” as well as the next question was not asked by the researcher when both parties (the child and the researcher) saw the content of the box on the table when she turned back to the child

^a^ the children considered as true-tellers

^b^ the children considered as liars

^c^the children considered as false-confessors

Of the peeking participants, 25 children directly confessed to peeking, but six children did not answer the first question but then confessed to knowing what was inside the box. Therefore, this group of 31 children (35%) was considered peekers–truth-tellers in our study. Moreover, only 12 children who peeked directly denied peeking (liars, according to Talwar & Lee, 2008), but 12 children also gave no answer to the first question and then, afterward, lied in response to the second question. Thus, 27% of peekers lied, and we consider them peekers–liars. Importantly, there was huge non-peeker group consisting of *n* = 65 false-confessors (who did not peek but answered ‘*yes*’ to the ‘*Did you peek*?’ question) and *n* = 60 truth-tellers (who did not peek and denied peeking). Interestingly, among the non-peekers, there were 14 children (9%) who probably accidently guessed what was inside the box.

#### Longitudinal consistency of inhibitory control

In Table 2 we present descriptive statistics for inhibitory control tasks at each time point, for the whole group of children.

**Table 2:** Descriptive statistics of inhibitory control tasks (SD in parentheses).

Time point	T2 (1.5 years of age, *n* = 151)	T3 (2 years of age, *n* = 175)	T4 (2.5 years of age, *n* = 105)
Mean time of delay in Snack Delay task in seconds	29.19 (24.63)	64.13 (34.83)	-
Mean number of correctly inhibited trials in Go/No Go Task	-	-	3.08 (1.87)

Although the mean times of delay at T2 and T3 correlated only marginally (*r* = .18, *p* = .068), the success rate (being able to wait until the end of the task) increased between these two time points, as presented elsewhere [38] and different developmental trajectories of the ability to delay gratification were distinguished (both stable and unstable).

### Inhibitory control of peekers and non-peekers

As predicted, non-peekers turned out to have better IC skills as they were able to wait longer than peekers in a delay of gratification task at 2 years old (*F*_*(1*,*174)*_ = 25.09, *p* < .001, η^2^ = .13; for 1.5 years *F*_*(1*,*150)*_ = .75, *p* = .39, η^2^ = .005) (see Table 3 for descriptive statistics). Moreover, they were also able to inhibit their response correctly in more trials during the Go/No-Go Task at 2.5 years of age (*F*_*(1*,*104)*_ = 10.79, *p* = .001, η^2^ = .10) (see Table 3 for descriptive statistics).

**Table 3:** Descriptive statistics of inhibitory control tasks (SD in parentheses) in the Groups of peekers and non-peekers.

Group	Mean time of delay in Snack Delay task in seconds		Mean number of correctly inhibited trials in Go/No Go Task
	1.5 years of age	2 years of age	2.5 years of age
Peekers	31.51 (24.24)	47.02 (36.86)	2.30 (1.93)
	*n* = 54	*n* = 60	*n* = 37
Non-peekers	27.90 (24.87)	73.06 (30.23)	3.50 (1.72)
	*n* = 97	*n* = 115	*n* = 68

*Note*. Analysed are groups of peekers and non-peekers, their lying behaviour was not taken into account in this analysis.

The other way to check whether not only IC skills but also the development of IC skills is related to peeking behaviour in our mTRP is to ask whether the increase in the ability to wait in a Snack Delay Test over the period from 1.5–2 years old is an important characteristic of non-peekers but not of the peeking group. The ANOVA analysis revealed that the growth of the time of delay was higher in non-peekers (*M* = 44.75, *SD* = 33.36, *n* = 69) than in peekers (*M* = 16.67, *SD* = 42.03, *n* = 39; *F*_*(1*,*106)*_ *=* 14.58, *p* < .001, η^2^ = .12).

#### Inhibitory control and lying behaviour

To examine relations between inhibitory control and lying behaviour we compared lying and true-telling children among peekers, as well as false-confessing and true-telling children among non-peekers. Descriptive statistics for these groups are presented in Table 4.

**Table 4:** Descriptive statistics of inhibitory control tasks (*SD* in parentheses).

Group	Mean time of delay in Snack Delay task in seconds		Mean number of correctly inhibited trials in Go/No Go Task
	1.5 years of age	2 years of age	2.5 years of age
Peekers: Truth-tellers	43.57 (25.14)	47.79 (37.12)	2.46 (1.81)
	*n* = 21	*n* = 22	*n* = 13
Peekers: Liars	28.38 (25.22)	44.22 (35.04)	2.36 (2.20)
	*n* = 15	*n* = 19	*n* = 11
Non-peekers: False-confessors	28.54 (25.88)	65.25 (34.31)	3.44 (1.45)
	*n* = 41	*n* = 47	*n* = 25
Non-peekers: Truth-tellers	30.61 (25.56)	81.53 (22.84)	3.64 (1.70)
	*n* = 36	*n* = 40	*n* = 28

Comparing the groups of peekers–liars and peekers–truth-tellers, we did not find any differences in IC at 1.5 years of age (*F*_*(1*,*35)*_ = 3.19, *p =* .08, η^2^ = .09), 2 years (*F*_*(1*,*40)*_ = 0.10, *p* = .75, η^2^ = .003) or at 2.5 years of age (*F*_*(1*,*23)*_ = 0.01, *p* = .91, η^2^ = .001).

As we were also interested in the characteristics of the group of non-peekers, we compared the results of IC tasks in non-peekers–false-confessors with non-peekers–truth-tellers. A significant difference in the ability to delay gratification at 2 years of age was found (*F*_*(1*,*86)*_ = 6.54, *p* < .05, η^2^ = .07), as children who falsely confessed in the mTRP were able to wait for a shorter amount of time than children who did not peek and told the truth. No other significant differences between the groups were found.

The last analysis used was a multinomial logistic regression to assess the role of IC in peeking and lying behaviours. As the previous analyses did not reveal any differences between the groups regarding IC at age 1.5, we used only the results in IC at age 2 (success in the IC task, or waiting till the end of the supposed time of delay, vs. failure, or waiting for the shorter amount of time) as predictors of belonging to one of the following groups: peekers–liars, peekers–truth-tellers, non-peekers–false-confessors and non-peekers–truth-tellers (reference group).

The model for the IC of 2-year-olds was statistically significant, χ^2^ = 15.83, *p* = .001. IC was a significant predictor of children’s peeking and lying behaviours, *Nagelkerke’s R*^*2*^ = .13). Specifically, the *Exp(B)* values in this analysis revealed that the 2-year-olds’ failure in the delay of gratification task increased the chance of peeking–truth-telling by more than 6 times (*Exp(B)* = 6.43), the chance of peeking–lying by more than 6 times (*Exp(B)* = 6.50) and the chance of non-peeking–false-confessing by more than 2 times (*Exp(B)* = 2.42), when compared to non-peeking–truth-telling behaviours.

## Discussion

The main aim of our study was to assess children’s lying behaviour in relation to their inhibitory control skills, using modified Temptation Resistance Paradigm and age-sensitive measures of inhibitory control. Thus, this study may be regarded as a conceptual replication of Evans and Lee [8] study, while acknowledging several modifications that we discuss in detail below.

First, and contrary to our expectation, most Polish 2.5-year-olds did not peek (65% non-peekers vs. 35% peekers). This is an important and novel result as, in comparison to previous studies (e.g.[8]; *N* = 65; M_age_ = 36 months, range: 25–48 months), we tested a large group of 2.5-year-old toddlers with a narrow age range (*N* = 252; *M* = 30 months, *SD* = 1week). Due to this characteristic of our group, our results show that most 2.5-year-old toddlers followed the adults’ rule in the mTRP, irrespective of the parent’s presence. One possible explanation for these striking differences relates to procedural changes in the TRP; however, it should be noted, that these changes (e.g. the presence of the parent) do not account completely for the observed result, as no differences were found between children who stayed in the room alone or with parent present. Therefore, one may relate this result also to cultural factors. It was shown that Canadian toddlers scored higher in social initiative than Chinese toddlers [8, 39]. Unfortunately, we are unaware of a similar comparison related to Polish children. However, in relation to early triadic interactions, we found [40] that compared to a US sample, Polish infants responded to joint attention bids and social interaction more often and initiated social interaction less often. Taken together with the results of the meta-analysis [41], which has shown that Poles are less individualistic compared to United States and Canada samples, this tendency to follow social rules may preliminarily explain why Polish children peeked less often.

Second, as expected, 2.5-year-old non-peeking children were less impulsive than peeking children. Non-peekers presented higher levels of IC, not only concurrently measured by the Go/No Go Task, but also their ability to inhibit was already present at the age of 2 as they were able to wait longer than peekers in the delay of gratification task. Moreover, those children who developed their ability to delay over the second half of the second year were more likely to be non-peekers than peekers at the age of 2.5. It should be noted, that the tasks we used to measure IC were different than measures used in the Evans and Lee study [8], but were developmentally sensitive [6]. These results are important, and they add new knowledge about the role of IC skills in resisting temptation in a paradigm that is widely used to measure not so much peeking but rather the first of all deceptive behaviours of toddlers. As we expected, non-peekers, who followed the rule and did not transgress it during the prohibition trial, were the ones who could delay receiving a favourite snack or who could inhibit spontaneous reactions. Thus, both early simple IC skills and later complex skills turned to be important determinants of non-peeking behaviours. In general, we claim that 2.5-year-old non-peekers, in comparison to peekers, are able to follow the adult’s rule due to their higher IC skills, which develop earlier and manifest with age-sensitive tasks.

Third, this main result and its interpretations are consistent with three other results we obtained. First, as in previous research (e.g. [8]), we found that only 27% of 2.5-year-old peeking children lied. In reference to the entire tested group, this was only 9% of children. Second, among the non-peeking children, we also found a large number of children (40%) who falsely confessed that they had peeked. Formally, we may call them liars as they admitted a false statement. However, a leaner interpretation of this behaviour would be that they were biased to answer ‘*yes*’ to the adult’s question: ‘*Did you peek*?’ Below, we discuss this interpretation in detail. Third, we must add that the findings of this interesting group of children are also consistent with the fact that non-peekers–false-confessors were more impulsive at age 2 than non-peekers–truth-tellers. Moreover, the multinomial logistic regression analysis revealed that failing the delay of gratification task at age 2 increased the chance of peeking in the mTRP at age 2.5 by more than 6 times and the chance of non-peeking but falsely confessing by more than 2 times, when compared to non-peeking–truth-telling behaviour. In other words, generally, when we refer to our results, the highest level of IC, at least at age 2, was presented by those 2.5-year-olds who followed the adult’s restrictions and were then able to tell the truth about their behaviour. All other three groups of children—those who did not peek and confessed they had, those who peeked and lied and those who peeked and then told the truth—were more impulsive than the rule following truth-tellers. Importantly, as we expected, there was no difference between peekers–liars and peekers–truth-tellers in their IC skills as measured at earlier ages (i.e. at the age of 1.5 and the age of 2 years) and concurrently (i.e. at the age of 2.5 years). In comparison to previous studies [8, 10, 31] that found such differences, we tested inhibitory skills longitudinally, not cross-sectionally, and with age-sensitive tasks. Thus, we believe that this study provides more contextually reliable and valid data about the role of inhibitory skills, which are measured with a TRP in children as young as 2.5 years old.

Moreover, on the one hand, the presented results seem consistent from a methodological point of view: among the 2.5-year-olds who are able to resist the temptation of peeking are rule followers, and among them, there were almost the same number of those who ‘lied’ and who admitted the truth (65 vs. 60). Nevertheless, those who were non-peekers–truth-tellers had the best IC skills. This result supports our idea that the TRP predominantly measures inhibition and not so much spontaneous lying in 2.5-year-olds. On the other hand, from a theoretical point of view, following adults’ expectations and instructions is an important toddler skill observed in 2.5-year-olds and is observed in many kinds of child–adult interactions. Such skills possibly build on earlier, already developed social-cognitive abilities such as joint attention and joint actions [42]. Such a possibility should be investigated in future studies.

Lastly, as far as our exploratory question is concerned—whether all non-peekers truly admit they peek or not—we found that as much as 40% of non-peeking children falsely confessed they had peeked. This result is extremely interesting. We believe this could be a clear demonstration of a ‘*yes*’ bias [32, 33]. Such young children react spontaneously and typically agree with adults. Fritzley and colleagues [33] suggest that when such young children are asked ‘*did you*’ questions, they confirm they understand and remember they should or should not do something. We might also state that with 2.5-year-olds, our results are consistent with previous results (see [43]), which demonstrated that a strong ‘*yes*’ bias is related to low IC in older children, i.e. 4-year-olds. In fact, the children who did not peek presented a higher ability to inhibit than the peeking children. In other words, non-peekers (and among them, non-peekers–truth-tellers) were in fact the least impulsive group.

This result also corroborates the idea that in general, in the TRP, such young children as 2.5 years old may react rather automatically, sometimes answering ‘*yes*’ to the ‘*Did you peek*?’ question after non-peeking behaviour and sometimes answering ‘*no*’ after the peeking behaviour. Thus, denying peeking in 2.5-year-olds may not be deliberate lying and this may be the reason for the weaker relation between children’s EF and their first lies than maintaining these lies [37]. In other words, so-called primary lies [9], as measured by the modified TRP, are probably, rather, pseudo-lies [27] or not truly deliberate instances of deceitfulness [28, 30]. Although this study provides unique results in that we followed a longitudinal research design with a rarely tested group of Polish 2.5-year-olds, we must admit some limitations of our study that are important for future studies. First, our modified version of the TRP provided interesting results but still differed from previous studies in the testing procedure and coding. We cannot exclude the possibility that bending down to look through the slot is more demanding that turning back or looking over the shoulder as in the original TRP. Also coding the answer to the second question in such young peeking children not as an index of semantic leakage control but as an index of lying is still disputable Thus, comparing our results with those of previous studies should be done cautiously. Second, we are very surprised that 10% of our non-peeking children accidently guessed that the bear was in the box during the prohibition trial. We cannot exclude the possibility that they just chose another very popular toy puppet after they saw the dog and the cat during the first two trials. Third, if the idea that a ‘*yes*’ bias in the mTRP is valid and if the toddlers’ answers in the mTRP were quick and thus spontaneous, in future studies, one should measure the time of children’s reactions after the question ‘*Did you peek*?’ The longest time should be observed in non-peekers–truth-tellers as the least impulsive group, and the shortest time is probably in peekers–liars. This would be more direct proof that such denying of peeking in the TRP in 2.5-year-olds is not the expression of deliberate lies but rather different levels of IC skills. Further research is thus needed on young children’s lying.

To sum up, this study highlights that 2.5-year-old children in a modified TRP reacted spontaneously. That is, if they peeked (35% of the group), they spontaneously—rather than deliberately—lied, and if they did not peek, they are as much prone to lie because of a ‘*yes*’ bias as they are able to tell the truth. Only those who were non-peekers and truth-tellers, i.e. rule followers, were the least impulsive. Thus, we cautiously claim that the deliberateness of early, or primary, lies is disputable. We conclude that to learn more about the early signs of deliberate deception in children younger than 3 or 4, future studies should include more comprehensive and culturally sensitive measures, such as not only the TRP, or modified TRP, but also other paradigms like deceptive pointing [34] or natural observations [1].
